# ZJU index: a novel model for predicting nonalcoholic fatty liver disease in a Chinese population

**DOI:** 10.1038/srep16494

**Published:** 2015-11-16

**Authors:** Jinghua Wang, Chengfu Xu, Yunhao Xun, Zhenya Lu, Junping Shi, Chaohui Yu, Youming Li

**Affiliations:** 1 Department of Gastroenterology, the First Affiliated Hospital, College of Medicine, Zhejiang University, Hangzhou 310003, China; 2 Department of Liver Diseases, Xixi Hospital of Hangzhou, Hangzhou, Zhejiang, China; 3 Department of Internal Medicine, the First Affiliated Hospital, College of Medicine, Zhejiang University; 4 Department of Liver Diseases, The Affiliated Hospital of Hangzhou Normal University, Hangzhou, China

## Abstract

Non-alcoholic fatty liver disease (NAFLD) is an important health issue worldwide. We aimed to develop a simple model to determine the presence of NAFLD in a Chinese population. A cross-sectional study with 9602 subjects was conducted. Potential predictors were entered into a stepwise logistic regression analysis to obtain the model. We used 148 patients with liver biopsy to validate this model. The model, named the ZJU index, was developed based on body mass index (BMI), fasting plasma glucose (FPG), triglycerides (TG), and the serum alanine aminotransferase (ALT) to serum aspartate transaminase (AST) ratio. The area under the receiver operating characteristic curve (AUROC) of the ZJU index to detect NAFLD was 0.822. At a value of <32.0, the ZJU index could rule out NAFLD with a sensitivity of 92.2%, and at a value of >38.0, the ZJU index could detect NAFLD with a specificity of 93.4%. In patients with liver biopsy, the ZJU index could detect steatosis with good accuracy, with an AUROC of 0.896. This study revealed that the ZJU index is a helpful model to detect NAFLD for community physicians in China. It was validated not only by a validation cohort but also by pathological data.

Non-alcoholic fatty liver disease (NAFLD) is an important issue for global public health in the twenty-first century^1^,^2^. Multiple measurements showed that there is an estimated worldwide prevalence of NAFLD ranging from 6% to 35%, with a median of 20% in the general public^3^. The disease affects 15% to 20% of adults in China, and continues to increase due to the pandemic of overweight and obesity in the Chinese population^4^. NAFLD covers a spectrum of liver diseases ranging from simple steatosis to non-alcoholic steatohepatitis (NASH) with various degrees of fibrosis that can eventually develop into cirrhosis^5^,^6^,^7^. Simple steatosis is considered to be benign, with a slow progression over many years, whereas NASH may progress to cirrhosis and hepatocellular carcinoma^8^,^9^,^10^,^11^.

Liver biopsy is the gold standard for the diagnosis of NAFLD^12^. However, the drawbacks of liver biopsy are its invasiveness, costliness and poor acceptance by patients. It is also not suitable as a screening test or as a risk assessment for the general population. Recently, many serological markers were found to be able to differentiate simple steatosis from steatohepatitis, such as ferritin, high sensitivity C-reactive protein, interleukin-6 and cytokeratin 18^13^,^14^,^15^. Some of these were found to be able to identify the fibrosis stage, such as hyaluronic acid and type IV collagen^16^,^17^. To improve the deficiencies of single markers, a number of models have been developed and validated to differentiate NAFLD from controls or to differentiate simple steatosis from NASH. The fatty liver index (FLI) provides a quantitative estimate of liver steatosis ranging from 0 to 100. FLI <30 rules out steatosis while FLI ≥60 suggests hepatic steatosis^18^. This metric has shown good performance in the detection of NAFLD in several population studies^19^,^20^. Other models, such as the hepatic steatosis index (HSI), the NAFLD liver fat score (NAFLD-LFS), the visceral adiposity index (VAI) and the triglyceride × glucose (TyG) index were also efficient for screening NAFLD^21^,^22^,^23^,^24^. Meanwhile, the HAIR score, the SteatoTest and the NashTest were used to distinguish between NASH and simple steatosis in some clinical studies^25^,^26^,^27^. However, most of these models were developed based on Westerners, and the parameters may not be suitable for Chinese people. Unfortunately, China has not developed its own model system for the detection of NAFLD.

In this study, we aimed to develop a simple model to determine the presence of NAFLD based on anthropometric parameters and standard laboratory tests. We also validated the model in a validation cohort and in patients with liver biopsy.

## Methods

### Subjects

The subjects of this study were recruited from adults who had health exanimations at the International Health Care Center, the First Affiliated Hospital, College of Medicine, Zhejiang University during the year 2014. The participants who had alcohol consumption greater than 140 g/week for men and 70 g/week for women, or had a history of viral hepatitis, autoimmune hepatitis, or other forms of chronic liver disease were excluded. A total of 9602 participants (7078 men and 2524 women) with a mean (standard deviation) age of 47.97 (10.13) years were included in the final analysis.

The study was approved by the Ethics Committee of the First Affiliated Hospital, College of Medicine, Zhejiang University. Because of the observational nature of the study, we verbally informed all participants about the study; written informed consent was not required. The subject information was anonymized at collection and anonymized prior to analysis. All methods were performed in accordance with the approved guidelines.

### Clinical examinations

Clinical examinations were performed according to procedures described previously^28^,^29^. Standing height, body weight and waist circumference were recorded for all participants. Body mass index (BMI) was calculated as the body weight divided by the standing height squared. Systolic and diastolic blood pressure were measured by standard clinical procedures.

Fasting blood samples were collected for the analysis of biochemical variables and were never frozen. The variables included liver enzymes, lipids, glucose, and uric acid. All biochemical variables were measured using a Hitachi 7600 autoanalyzer (Hitachi, Tokyo, Japan) and standard methods.

### Diagnosis of NAFLD

NAFLD was diagnosed based on the criteria proposed by the Chinese Liver Disease Association^30^. Hepatic ultrasound examination was performed by a trained ultrasonographist who was blinded to clinical assessments and the results of the biochemical analysis. The hepatic ultrasound examination was performed using an ACUSON Sequoia 512 ultrasound machine with a 3.5-MHz probe (Siemens, Mountain View, CA).

### Histological assessment

A group of subjects with liver biopsy data were recruited at the First Affiliated Hospital, College of Medicine, Zhejiang University, Hangzhou Sixth People’s Hospital, and Ningbo Medical Treatment Center Lihuili Hospital. This study was approved by the Ethics Committee of the First Affiliated Hospital, College of Medicine, Zhejiang University, and all patients provided their written informed consent. Liver biopsies were performed due to unexplained abnormal liver function or due to suspected NAFLD during cholecystectomy for gallstone disease. Liver biopsies were fixed, paraffin-embedded, stained with hematoxylin–eosin, reticulin, and Masson trichrome stains. Liver histology was assessed independently by two experienced pathologists who were blinded to the clinical data.

Steatosis was categorized as none if the presence of steatosis was less than 5%, mild (≥5–33%), moderate (>33–66%) and severe (>66%). The NAFLD activity score (NAS)^31^ was used to define NASH; NAS≥5 corresponded to a diagnosis of “NASH”, NAS = 3–4 corresponded to “borderline NASH”, and NAS <3 corresponded to “not NASH”. The severity of fibrosis was expressed on a 4-point scale, as follows: 0 = none, 1 = perivenular and/or perisinusoidal fibrosis in zone 3, 2 = combined pericellular portal fibrosis, 3 = septal/bridging fibrosis, 4 = cirrhosis.

### Statistical analysis

Data were managed and analyzed using SPSS software version 17.0 (SPSS, Inc., Chicago, IL). Continuous variables were compared using the Mann–Whitney *U*-test, and categorical variables were compared using the chi-squared test. Analysis of variance (ANOVA) was used to assess the differences in liver histology grade. All variables except age and gender were evaluated as continuous predictors in univariate analysis. The main reason why we excluded age and gender in the univariate analysis was that the study participates were matched by age and gender. The variables with higher odds ratio (OR) were added to a multiple logistic regression model to identify independent predictors for the presence of NAFLD.

To identify candidate predictors of NAFLD, we performed a stepwise logistic regression analysis on 1000 bootstrap samples (probability to enter =0.05 and probability to remove =0.10)^32^. A simple model using representative variables was established to predict NAFLD based on the results of multiple logistic regression analysis. The goodness of fit of the models was evaluated using the Hosmer-Lemeshow statistic. The predictive accuracy of the models for detecting NAFLD or steatosis was evaluated using areas under receiver-operating characteristic curves (AUROCs) with 95% confidence intervals (CI). Sensitivities, specificities, positive likelihood ratios, and negative likelihood ratios of the model were also calculated. AUROC were compared using the Delong test. A *P*-value less than 0.05 was considered statistically significant.

## Results

### Clinical characteristics of the study participants

**Figure 1:**
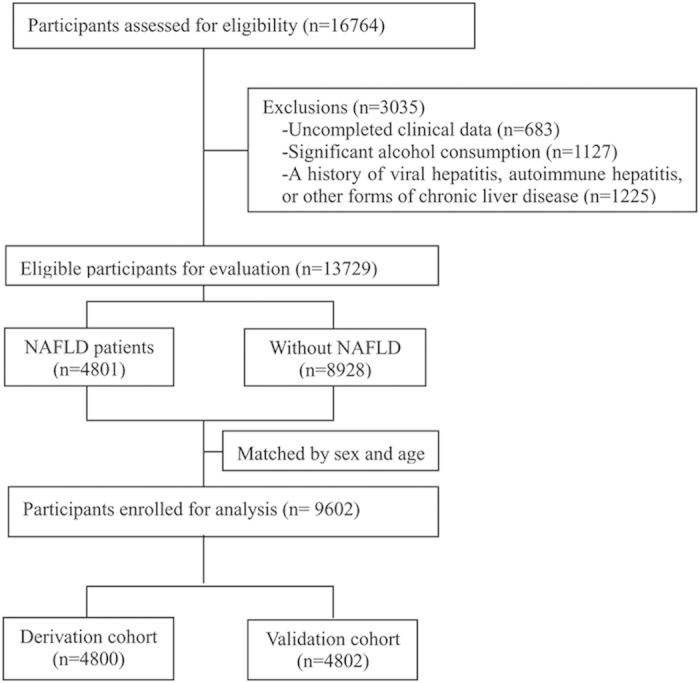
Inclusion and exclusion flow chart.

Of the 13729 participants eligible for evaluation, 4801 were diagnosed as having NAFLD by ultrasound examination. Among these 4801 subjects with NAFLD, 3539 (73.7%) were male, and the mean age was 48.0 years. The individuals without NAFLD were randomly selected among the remaining 8928 participants with 1:1 matching by sex and age (within 1 year). Finally, a total of 4801 pairs (9602 subjects) of cases and age- and sex-matched controls were randomly assigned to the derivation cohort (2400 pairs, 4800 subjects) and to the validation cohort (2401 pairs, 4802 subjects) (Fig. 1). No significant difference was found between these two cohorts in terms of clinical characteristics (Table 1).

### Fatty liver index and hepatic steatosis index in the study population

**Figure 2:**
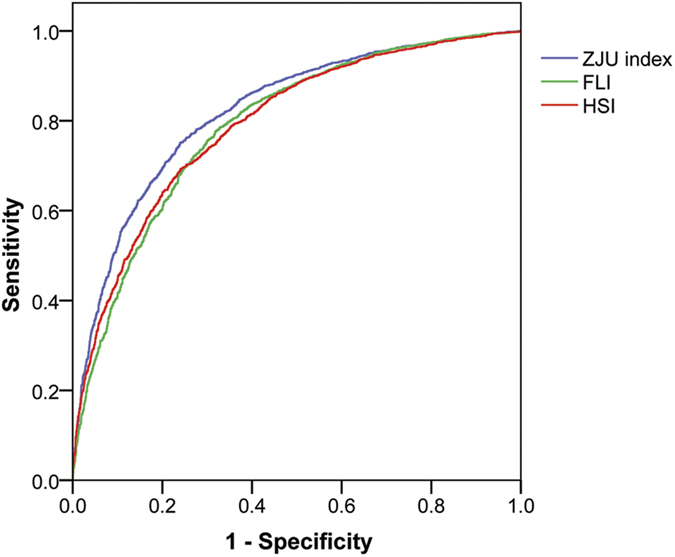
Receiver-operating characteristic (ROC) curve of FLI, HSI and ZJU index for detecting NAFLD. The area under the ROC curve of FLI, HSI, and ZJU index were 0.790 (95% CI: 0.778–0.803), 0.793 (95% CI: 0.781–0.806), and 0.822 (95% CI: 0.810–0.834), respectively. FLI: fatty liver index; HSI hepatic steatosis index.

We first validated FLI using the derivation cohort; median value of FLI was 36.9, and the AUROC of FLI for detecting NAFLD was 0.790 (95% CI: 0.778–0.803) (Fig. 2). A total of 2297 (47.9%) subjects were at a FLI value <30, with a sensitivity of 74.6% (95% CI, 72.9%–76.4%); 1086 (22.6%) subjects were at a FLI value of >60, with a specificity of 91.7% (95% CI, 90.6%–92.8%).

Median value of HSI was 33.8, and the AUROC of HSI for detecting NAFLD was 0.793 (95% CI: 0.781–0.806) (Fig. 2). A total of 1103 (23.6%) subjects were at a HSI value <30, with a sensitivity of 92.2% (95% CI, 91.1%–93.3%); 1486 (31.0%) subjects were at a HSI value of >36, with a specificity of 87.8% (95% CI, 86.5%–89.1%).

### Derivation of the ZJU index

A total of 2400 subjects with NAFLD and 2400 age- and sex-matched subjects without NAFLD were included in the derivation cohort. Table 2 gave the characteristics of the subjects with and without NAFLD in the derivation cohort. Univariate analysis showed that height, weight, BMI, waist circumference, systolic and diastolic blood pressure, heart rate, hemoglobin, white blood cells, platelets, albumin, globulin, fasting plasma glucose (FPG), triglycerides (TG), total cholesterol, high-density lipoprotein cholesterol (HDL-C), low-density lipoprotein cholesterol (LDL-C), very low-density lipoprotein cholesterol (VLDL-C), uric acid, alanine aminotransferase (ALT), aspartate aminotransferase (AST), the ALT/AST ratio, gamma-glutamyl transpeptidase (GGT), alkaline phosphatase (ALP), cholinesterase, alpha fetoprotein (AFP) and alpha fucosidase (AFU) were significantly different between cases and controls. Among these variables, significant interactions were found between height, weight, BMI and waist circumference; between AST, ALT and the ALT/AST ratio; and between total cholesterol, TG, HDL-C, LDL-C, and VLDL-C. To avoid these interactions, we incorporated representative variables with the highest ORs into the multivariate analysis. Finally, we utilized BMI, FPG, TG and the ALT/AST ratio for the multivariate analysis.

The multivariate analysis showed that BMI (OR: 1.373, 95% CI: 1.336–1.412; *P* < 0.001), FPG (OR: 1.255, 95% CI: 1.166–1.350; *P* < 0.001), TG (OR: 1.436, 95% CI: 1.335–1.544; *P* < 0.001) and the ALT/AST ratio (OR: 2.400, 95% CI: 1.985–2.901; *P* < 0.001) were independent risk factors for NAFLD after adjusting for interactions between variables. In this multiple logistic regression model, the probability of having NAFLD was e^−10.52+0.317×BMI+0.875×ALT-to-AST ratio+0.227×FPG +0.362×TG^/(1+ e^−10.52+0.317×BMI+0.875×ALT-to-AST ratio+0.227×FPG +0.362×TG^). We utilized the exponent of this formula and changed the multiplicative factors into approximate integers. In addition, to adjust for the difference in BMI between male and female subjects, we added 2 points to females. We call this formula the ZJU index as follows:


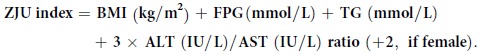


The AUROC of the original formula was 0.812 (95% CI: 0.800–0.824), and the AUROC of the ZJU index was 0.822 (95% CI: 0.810–0.834) in the derivation cohort (Fig. 2). This was significantly higher than that in FLI and HSI (*P* < 0.001). For males and females, the AUROC (95% CI) of the ZJU index was 0.817 (0.803–0.831) and 0.839 (0.817–0.861), respectively. At a value of <32.0, the ZJU index could rule out NAFLD with a sensitivity of 92.2% (95% CI: 91.1%–93.2%); and at a value of >38.0, the ZJU index could detect NAFLD with a specificity of 93.4% (95% CI: 92.4%–94.4%) (Table 3).

In the derivation cohort, 1242 subjects (25.8%) had a ZJU index <32 and 1178 subjects (24.5%) had a ZJU index >38. According to these cutoff values, 2073 subjects (85.7% of subjects with a ZJU index of <32 or >38) were correctly classified.

In subjects no more than 40 years old (n = 1095), the predictive values of the ZJU index were better. In the derivation cohort, the AUROC of the ZJU index was 0.867 (95% CI: 0.845–0.888). At a value of <32, the ZJU index could rule out NAFLD with a sensitivity of 91.2% (95% CI: 88.8%–93.5%), and at a value of >38, the ZJU index could detect NAFLD with a specificity of 95.8% (95% CI: 94.2%–97.4%).

### Validation of the ZJU index

The AUROC of the original formula was 0.817 (95% CI: 0.805–0.829); the AUROC of the ZJU index was 0.826 (95% CI: 0.815–0.838) in the validation cohort. For males and females, the AUROC (95% CI) of the ZJU index was 0.825 (0.811–0.838) and 0.831 (0.809–0.853), respectively. At a value of <32, the ZJU index could rule out NAFLD with a sensitivity of 92.4% (95% CI: 91.4%–93.5%); at a value of >38, the ZJU index could detect NAFLD with a specificity of 93.3% (95% CI: 92.2%–94.3%) (Table 3).

In the validation cohort, 1216 subjects (25.3%) had a ZJU index <32 and 1178 subjects (24.5%) had a ZJU index >38. According to these cutoff values, 2052 subjects (85.7% of subjects with a ZJU index of <32 or >38) were correctly classified.

In subjects no more than 40 years old (n = 1128), the predictive values of the ZJU index were better. In the validation cohort, the AUROC (95% CI) of the ZJU index was 0.867 (0.846–0.887). At a value of <32, the ZJU index could rule out NAFLD with a sensitivity of 91.7% (95% CI: 89.4%–94.0%); at a value of >38, the ZJU index could detect NAFLD with a specificity of 95.6% (95% CI: 94.0%–97.3%).

### Characteristics of the liver biopsy participants

We next assessed the ZJU index in liver biopsy subjects. Table S1 summarizes the baseline features of the study population. Of the 148 participants with liver biopsy, 119 (80.4%) were male and the mean age was 44.2 years. The median value of the ZJU index was 38.4 (25^th^–75^th^ quartile: 36.1–40.6). The data for histological evaluation was presented in Table S1.

**Figure 3:**
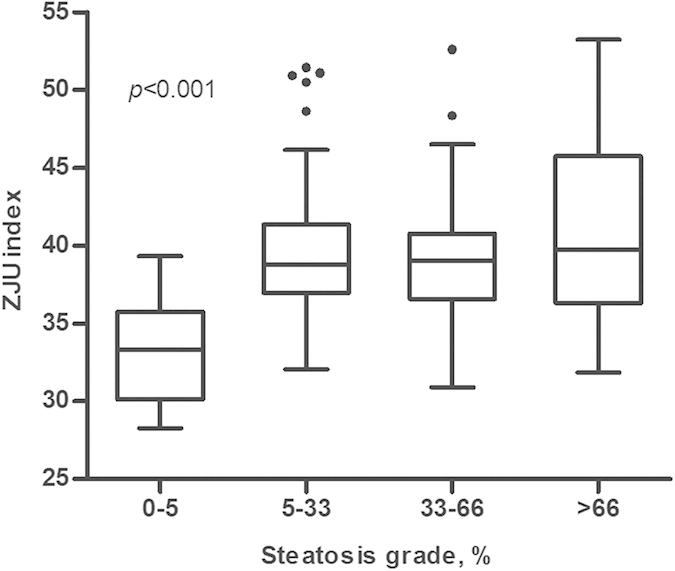
Distribution of biomarkers according to the histological grade of steatosis. The box represents the interquartile range. The line across the box indicates the median. The ‘whiskers’ extend from the box to the highest and lowest values, excluding outliers (black dots). The ZJU index in patients with steatosis was significantly higher than in the non-steatosis group (*P* < 0.001).

**Figure 4:**
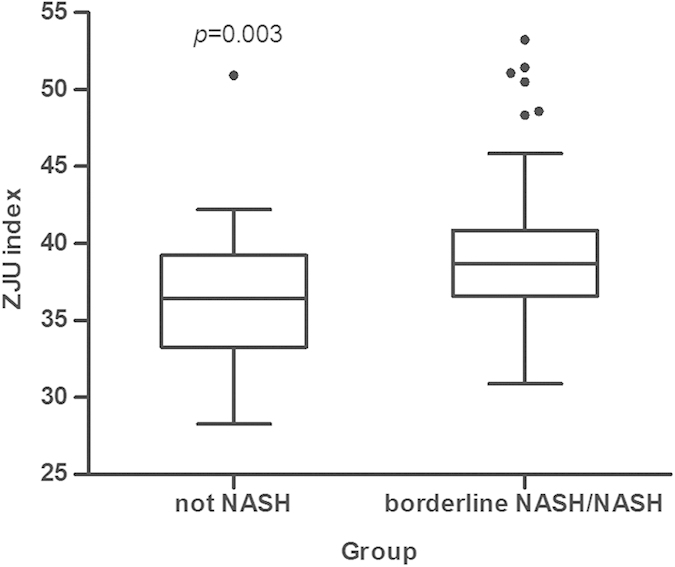
Distribution of steatosis biomarkers according to the histological grade of the NAS score. The box represents the interquartile range. The line across the box indicates the median. The ‘whiskers’ extend from the box to the highest and lowest values, excluding outliers (black dots). The ZJU index in patients with borderline NASH/NASH was significantly higher than in the non-NASH group (*P* = 0.003).

As shown in Fig. 3, The ZJU index in patients with steatosis was significantly higher than those without steatosis (*P* < 0.001). The AUROC (95% CI) of the ZJU index for detecting steatosis was 0.896 (0.818–0.974) (Figure S1). At a value of >38.0, the ZJU index could detect steatosis with a specificity of 93.3%. The ZJU index in patients with NASH/borderline NASH was significantly higher than the no NASH group (Fig. 4) (P = 0.003). However, there was no significant difference in the ZJU index among different fibrosis grades (Figure S2).

## Discussion

This study developed a simple model for predicting NAFLD in a Chinese people. The model used BMI and standard laboratory tests, including fasting plasma glucose, triglycerides, ALT and AST. This model performed better than FLI in the Chinese people, and we also found that this model performed better in subjects younger than 40 years old. The pathology results confirmed that this model can be used for the detection of steatosis.

Radiological imaging studies such as ultrasound, CT and MRI, have a good accuracy in the diagnosis of fatty liver. A recent meta-analysis showed that ultrasound is an accurate and reliable tool to detect moderate to severe fatty liver, with a specificity of 93.4% and a sensitivity of 84.8% to evaluate NAFLD^33^. Nevertheless, ultrasonography cannot distinguish NASH from simple steatosis, and it is dependent on the operator’s experience and the technological sophistication. Meanwhile, CT and MRI are too expensive to be used as routine screening tests. FLI is a biochemical assessment of steatosis that was proposed in Italy^18^. An FLI <30 rules out hepatic steatosis while an FLI ≥60 confirms hepatic steatosis. This metric has shown good performance in detecting NAFLD in several population studies^19^,^20^. FLI includes BMI, GGT, TG and waist circumference in its model. As we know, BMI and waist circumference are not as pronounced in Chinese compared to Caucasians^34^. We tried to revise FLI with appropriate coefficients for our population, but the AUROC of revised FLI is 0.806 (95% CI: 0.793–0.818) in the derivation cohort (supplementary formula). Therefore, FLI, which was established based on Italians, may not be suitable for the Chinese population.

In this study, we developed a novel model for the prediction of NAFLD. The AUROC of the ZJU index was 0.822 (95% CI: 0.810–0.834). This was better than FLI and HSI in the Chinese population (Fig. 2). This model can be used to select eligible subjects for further examination. When the ZJU index <32, patients were less likely to have a fatty liver. When the ZJU index >38, patients were more likely to have a fatty liver and the individual should then undergo a radiological imaging screen. We also verified this metric in patients with liver biopsy, and found that the ZJU index works as well as the gold standard. The ZJU index can predict the presence of steatosis with an AUROC of 0.896 (95% CI: 0.818–0.974). When the ZJU index >38, the ZJU index could detect steatosis with a specificity of 93.3%. We also found that the ZJU index of patients with non-NASH was significantly lower than patients with borderline NASH or NASH (Fig. 4). This observation indicated that the ZJU index may also be helpful for distinguishing NASH from simple steatosis.

It was reported that there was a significant and continuous increase in the prevalence of obesity in children and adolescents in last 20 years^35^. Another study from the north of China drew a similar conclusion^36^. This tendency was also found in diabetes in China according to national epidemiological studies^37^,^38^. Both obesity and diabetes are risk factors for NAFLD^39^,^40^,^41^. The ZJU index in our study performed better in subjects younger than 40, as the AUROC was 0.867 (95% CI, 0.845–0.888). The ZJU index makes it possible for young people in China to diagnose NAFLD at an early stage. Because it was a simple sum of BMI, FPG and TG, BMI seemed more weight than others. As the standard deviation of these variables did not differ so much, the simple obesity might not affect the results. Another issue is that that the use of medications for diabetes and dyslipidemia may impact FPG and TG levels. In this study, unfortunately, the medical history of participants at baseline was not available. This may affect the FPG and TG level in our study to some extent. Future studies will need to assess the impact of medical history on the ZJU index.

In conclusion, our study developed a simple model to predict the presence of NAFLD in the Chinese population. This model can be used as a simple, noninvasive, and cost-effective tool for screening NAFLD in Chinese.

## Additional Information

**How to cite this article**: Wang, J. *et al.* ZJU index: a novel model for predicting nonalcoholic fatty liver disease in a Chinese population. *Sci. Rep.* **5**, 16494; doi: 10.1038/srep16494 (2015).

## Supplementary Material

Supplementary Information

## Figures and Tables

**Table 1:** Clinical characteristics of the derivation and validation cohorts.

	Derivation cohort(n = 4800)	Validation cohort(n = 4802)	*P*
Age (years)	48.0 ± 10.2	47.9 ± 10.1	0.674
Sex (male%)	73.6	73.6	0.981
Height (cm)	166.60 ± 7.87	166.56 ± 7.93	0.989
Weight (kg)	68.30 ± 11.24	68.33 ± 11.16	0.888
BMI (kg/m^2^)	24.53 ± 3.18	24.55 ± 3.13	0.756
Waist circumference (cm)	86.32 ± 9.15	86.37 ± 9.13	0.806
SBP (mmHg)	129.30 ± 18.00	128.89 ± 17.56	0.264
DBP (mmHg)	79.24 ± 11.53	79.13 ± 11.24	0.631
Heart rate (per min)	74.21 ± 10.65	74.16 ± 10.68	0.848
Hemoglobin (g/L)	149.75 ± 15.14	149.97 ± 15.68	0.487
Platelet count (×10^9^)	206.11 ± 51.04	204.46 ± 50.61	0.110
White blood cell count (×10^9^)	6.04 ± 1.54	6.01 ± 1.53	0.364
Neutrophil count (×10^9^)	3.45 ± 1.16	3.42 ± 1.15	0.235
Albumin (g/L)	46.42 ± 3.13	46.33 ± 3.14	0.186
Globulin (g/L)	26.25 ± 3.41	26.25 ± 3.42	0.963
Uric acid (μmol/L)	348.91 ± 86.99	347.62 ± 85.55	0.463
Fasting plasma glucose (mmol/L)	5.10 ± 1.12	5.11 ± 1.23	0.604
Triglycerides (mmol/L)	1.69 ± 1.33	1.72 ± 1.45	0.388
Total cholesterol (mmol/L)	4.79 ± 0.88	4.82 ± 0.92	0.176
LDL-C (mmol/L)	2.64 ± 0.67	2.65 ± 0.68	0.320
VLDL-C (mmol/L)	0.94 ± 0.54	0.96 ± 0.56	0.107
HDL-C (mmol/L)	1.21 ± 0.30	1.21 ± 0.30	0.268
AFU (IU/L)	27.58 ± 7.79	27.55 ± 7.71	0.858
ALT (IU/L)	25.94 ± 22.40	26.44 ± 7.76	0.333
AST (IU/L)	22.87 ± 11.49	23.18 ± 16.07	0.281
GGT (IU/L)	39.41 ± 59.43	39.34 ± 46.94	0.948
Cholinesterase (IU/L)	8992 ± 1651	9011 ± 1651	0.577
ALP (IU/L)	67.80 ± 19.64	68.08 ± 19.43	0.477
AFP (ng/ml)	2.96 ± 1.72	3.07 ± 3.34	0.051

Data are expressed as the mean ± SD. BMI: body mass index; SBP: systolic blood pressure; DBP: diastolic blood pressure; LDL-C: low-density lipoprotein cholesterol; VLDL-C: very low-density lipoprotein cholesterol; HDL-C: high-density lipoprotein cholesterol; ALT: alanine aminotransferase; AST: aspartate aminotransferase; GGT: gamma-glutamyl transpeptidase; AFU: alpha fucosidase; ALP: alkaline phosphatase; AFP: alpha fetoprotein.

**Table 2:** Case and age- and sex-matched control data in the derivation cohort.

	Control (n = 2400)	NAFLD (n = 2400)	P
Age (years)	48.0 ± 10.2	48.1 ± 10.1	0.798
Sex (male%)	73.7	73.7	1.000
Height (cm)	166.54 ± 7.83	166.65 ± 7.91	<0.001
Weight (kg)	64.03 ± 9.87	72.57 ± 10.91	<0.001
BMI (kg/m^2^)	23.04 ± 2.67	26.02 ± 2.95	<0.001
Waist circumference (cm)	82.32 ± 8.25	90.32 ± 8.21	<0.001
SBP (mmHg)	125.66 ± 17.63	132.93 ± 17.63	<0.001
DBP (mmHg)	76.80 ± 11.57	81.68 ± 10.96	<0.001
Heart rate (per min)	73.05 ± 10.47	75.36 ± 10.71	<0.001
Hemoglobin (g/L)	148.19 ± 15.07	151.32 ± 15.06	<0.001
Platelet count (×10^9^)	200.57 ± 48.61	211.66 ± 52.79	<0.001
White blood cell count (×10^9^)	5.76 ± 1.50	6.32 ± 1.52	<0.001
Neutrophil count (×10^9^)	3.30 ± 1.15	3.60 ± 1.16	<0.001
Albumin (g/L)	46.18 ± 3.17	46.65 ± 3.07	<0.001
Globulin (g/L)	25.98 ± 3.35	26.53 ± 3.44	<0.001
Uric acid (μmol/L)	330.35 ± 82.46	367.47 ± 87.45	<0.001
Fasting plasma glucose (mmol/L)	4.88 ± 0.84	5.31 ± 1.31	<0.001
Triglycerides (mmol/L)	1.33 ± 0.89	2.06 ± 1.58	<0.001
Total cholesterol (mmol/L)	4.69 ± 0.86	4.89 ± 0.89	<0.001
LDL-C (mmol/L)	2.60 ± 0.65	2.68 ± 0.68	<0.001
VLDL-C (mmol/L)	0.81 ± 0.42	1.08 ± 0.61	<0.001
HDL-C (mmol/L)	1.29 ± 0.31	1.14 ± 0.28	<0.001
AFU (IU/L)	26.61 ± 7.56	28.56 ± 7.90	<0.001
ALT (IU/L)	21.49 ± 21.31	30.39 ± 22.58	<0.001
AST (IU/L)	21.64 ± 11.57	24.10 ± 11.27	<0.001
ALT/AST ratio	0.95 ± 0.33	1.20 ± 0.43	<0.001
GGT (IU/L)	32.66 ± 68.24	46.15 ± 48.14	<0.001
Cholinesterase (IU/L)	8510 ± 1616	9474 ± 1541	<0.001
ALP (IU/L)	66.66 ± 19.31	68.93 ± 19.91	<0.001
AFP (ng/ml)	3.03 ± 1.88	2.90 ± 1.54	0.007

Data are expressed as the mean ± SD. NAFLD: Nonalcoholic fatty proselytizing liver disease; SD: standard deviation; BMI: body mass index; SBP: systolic blood pressure; DBP: diastolic blood pressure; LDL-C: low-density lipoprotein cholesterol; VLDL-C: very low-density lipoprotein cholesterol; HDL-C: high-density lipoprotein cholesterol; ALT: alanine aminotransferase; AST: aspartate aminotransferase; GGT: gamma-glutamyl transpeptidase; AFU: alpha fucosidase; ALP: alkaline phosphatase; AFP: alpha fetoprotein.

**Table 3:** Diagnostic accuracy of the ZJU index.

Derivation cohort				
Cut-offpoint	Sensitivity(%)	Specificity(%)	LR+	LR−
≥30	96.9	23.0	1.26	0.13
≥31	95.4	32.5	1.41	0.14
**≥32**	**92.2**	**43.9**	**1.64**	**0.18**
≥33	87.9	56.5	2.02	0.21
≥34	81.4	66.6	2.44	0.28
≥35	73.9	76.8	3.18	0.34
≥36	62.9	84.6	4.09	0.44
≥37	52.8	89.9	5.21	0.53
**≥38**	**42.5**	**93.4**	**6.41**	**0.62**
≥39	33.3	95.8	7.82	0.70
≥40	24.8	97.2	8.88	0.77
Validation cohort				
≥30	98.0	23.4	1.28	0.09
≥31	95.7	32.9	1.43	0.13
**≥32**	**92.5**	**43.2**	**1.63**	**0.17**
≥33	88.5	56.8	2.05	0.20
≥34	82.4	67.2	2.52	0.26
≥35	74.4	75.9	3.09	0.34
≥36	63.9	84.3	4.07	0.43
≥37	52.5	90.1	5.30	0.53
**≥38**	**42.3**	**93.3**	**6.27**	**0.62**
≥39	32.9	95.8	7.91	0.70
≥40	24.8	97.1	8.62	0.77

LR+: positive likelihood ratio; LR−: negative likelihood ratio.
