# Supplementary material for: ZJU index: a novel model for predicting nonalcoholic fatty liver disease in a Chinese population
Source: Sci Rep. 2015 Nov 16;5:16494. doi: 10.1038/srep16494 (PMC4645098; doi:10.1038/srep16494)
Supplement: Supplementary Information [file srep16494-s1.pdf]

ZJU index: a novel model for predicting nonalcoholic fatty liver disease in a Chinese population

Jinghua Wang[1], Chengfu Xu[1], Yunhao Xun[2], Zhenya Lu[3], Junping Shi[4], Chaohui Yu[1,*], and Youming Li[1,*]

[1] Department of Gastroenterology, the First Affiliated Hospital, College of Medicine, Zhejiang University, Hangzhou 310003, China

[2] Department of Liver Diseases, Xixi Hospital of Hangzhou, Hangzhou, Zhejiang, China

[3] Department of Internal Medicine, the First Affiliated Hospital, College of Medicine, Zhejiang University

[4] Department of Liver Diseases, The Affiliated Hospital of Hangzhou Normal University, Hangzhou, China

[*] **Correspondence:**

Prof. Youming Li, or Dr. Chaohui Yu, Department of Gastroenterology, the First Affiliated Hospital, College of Medicine, Zhejiang University. 79 Qingchun Road, Hangzhou 310003, P. R. China. Tel: +86-571-87236603; Fax: +86-571-87236611

E-mails: fufu715@hotmail.com (Y.L.), or ych623@sina.com (C.Y.)

† Jinghua Wang, Chengfu Xu and Yunhao Xun contributed equally to this work.

Table S1: Clinical characteristics of the liver biopsy participants

Age (years)	44.2 (11.2)
Males, n (%)	119(80.4)
Body mass index (kg/m^2)	25.62(23.93-27.22)
Fasting blood sugar (mmol/L)	5.13(4.71-5.59)
Triglycerides (mmol/L)	1.85(1.46-2.55)
ALT (IU/L)	79(46-127.5)
AST (IU/L)	49(32-75.5)
ZJU index	38.36(36.08-40.64)
Histological data	
Steatosis	
None (<5%),	15(10.1)
Mild (5–33%),	69(46.6)
Moderate (33–66%),	53(35.8)
Severe (>66%),	11(7.4)
NAS[a]	
Simple Steatosis(NAS=0-2)	36(25.9)
Borderline Group(NAS=3-4)	48(34.5)
NASH(NAS=5-7)	55(39.6)
Fibrosis stage[a]	
0	57(41.0)
1	46(33.1)
2	17(12.2)
3	15(10.8)
4	4(2.9)

Data are expressed as median (IQR) and as frequency (percentage).

ALT: alanine aminotransferase; AST: aspartate aminotransferase; NAS:NAFLD activity score

[a] Nine subjects had no data of NAFLD activity score and fibrosis stage.

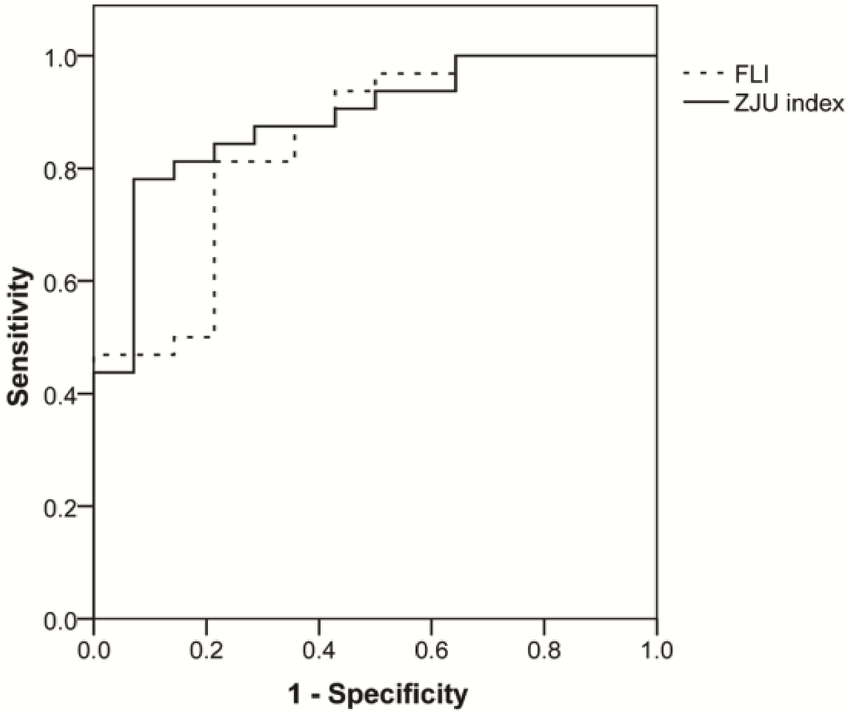

Figure S1. Receiver-operating characteristic (ROC) curve of ZJU index and FLI for detecting steatosis. The AUROC of ZJU index for detecting steatosis was 0.896 (95%CI: 0.818-0.974). The AUROC of FLI for detecting steatosis was 0.844 (95%CI: 0.720–0.967).

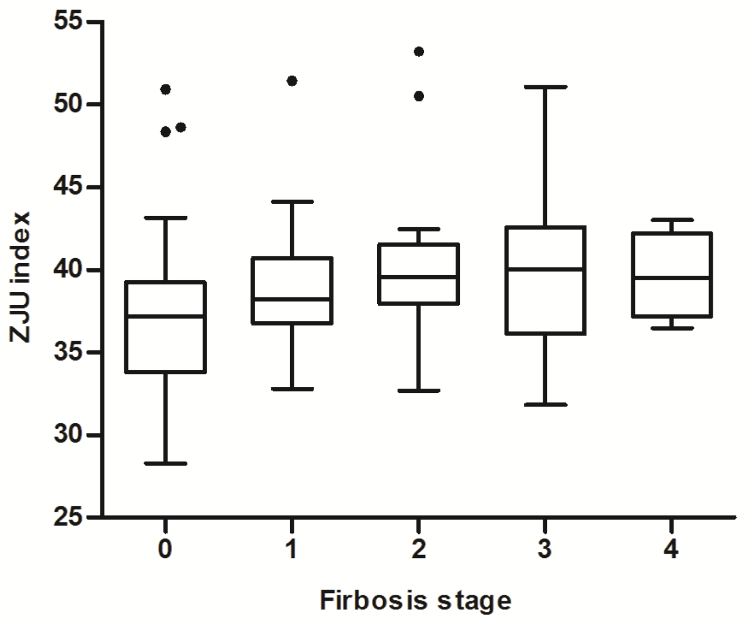

Figure S2. Distribution of steatosis biomarkers according to the histological grade of Firbosis stage. The box represents the interquartile range. The line across the box indicates the median. The 'whiskers' extend from the box to the highest and lowest values, excluding outliers (black dots). There was no significant difference of ZJU index in each fibrosis stage

Supplementary formula

We tried to revise the coefficient of FLI adapt to our population

The formula of revised FLI is:

$e^{0.836*loge(TG)+0.295*BMI+0.197*loge(GGT)+0.017*WC-13.428}/(1+e^{0.836*loge(TG)+0.295*BMI+0.197*loge(GGT)+0.017*WC-13.428})*100$

TG: mg/dl, BMI: (kg/m^2), GGT: (IU/L), WC: cm

The AUROC of revised FLI is 0.806 (95%CI: 0.793 – 0.818) in the derivation cohort.
